# Comparisons between mild and severe cases of hand, foot and mouth disease in temporal trends: a comparative time series study from mainland China

**DOI:** 10.1186/s12889-016-3762-x

**Published:** 2016-10-21

**Authors:** Xiong Xiao, Qiaohong Liao, Michael G. Kenward, Yaming Zheng, Jiao Huang, Fei Yin, Hongjie Yu, Xiaosong Li

**Affiliations:** 1 Department of Epidemiology and Biostatistics, West China School of Public Health, Sichuan University, Chengdu, China; 2 Department of Medical Statistics, London School of Hygiene and Tropical Medicine, London, UK; 3 Division of Infectious Disease & Key Laboratory of Surveillance and Early Warning on Infectious Disease, Chinese Centre for Disease Control and Prevention, Beijing, China

**Keywords:** Enterovirus infection, Hand, foot and mouth disease, Case surveillance, Comparative time series analysis, Mainland China

## Abstract

**Background:**

Over recent decades, hand, foot and mouth disease (HFMD) has emerged as a serious public health threat in the Asia-Pacific region because of its high rates of severe complications. Understanding the differences and similarities between mild and severe cases can be helpful in the control of HFMD. In this study, we compared the two types of HFMD cases in their temporal trends.

**Methods:**

We retrieved the daily series of disease counts of mild and severe HFMD cases reported in mainland China in the period of 2009–2014. We applied a quasi-Poisson regression model to decompose each series into the long-term linear trend, periodic variations, and short-term fluctuations, and then we compared each component between two series separately.

**Results:**

A total of 11,101,860 clinical HFMD cases together with 115,596 severe cases were included into this analysis. We found a biennial increase of 24.46 % (95 % CI: 22.80–26.14 %) for the baseline of disease incidence of mild cases, whereas a biennial decrease of 8.80 % (95 % CI: 7.26–10.31 %) was seen for that of severe cases. The periodic variations of both two series could be characterized by a mixture of biennial, annual, semi-annual and eight-monthly cycles. However, compared to the mild cases, we found the severe cases vary more widely for the biennial and annual cycle, and started its annual epidemic earlier. We also found the short-term fluctuations between two series were still significantly correlated at the current day with a correlation coefficient of 0.46 (95 % CI: 0.43–0.49).

**Conclusions:**

We found some noticeable differences and also similarities between the daily series of mild and severe HFMD cases at different time scales. Our findings can help us to deepen the understanding of the transmission of different types of HFMD cases, and also provide evidences for the planning of the associated disease control strategies.

## Background

Hand, foot and mouth disease (HFMD) is a common childhood infectious disease caused by viruses that belong to the Enterovirus group, mainly by Coxsackievirus A16 (CVA 16), Enterovirus 71 (EVA 71) and, more recently, Coxsackievirus A6 (CVA 6) [1]. It is typically characterized by a febrile illness followed by rash on hands and feet, sometimes also with vesicular/ulcer in the mouth [2]. In most cases, the disease is mild and self-limiting and requires no more treatment other than symptomatic relief [3]. However, some patients, especially those infected with EVA 71, may develop severe complications involving the central nervous system (CNS) such as meningitis, encephalitis and acute flaccid paralysis [4, 5]. A few patients can even progress to fatal cardiopulmonary failure.

Following the first observation of the clinical syndrome of HFMD in 1957 in New Zealand [6], HFMD and associated severe CNS disease have been reported globally [7–13]. The outbreaks of HFMD reported before the mid-1990s are normally considered as “benign” with rare severe and fatal cases, or many severe cases have been initially misdiagnosed as some other diseases (like poliomyelitis or encephalitis) [2]. However, over the last few decades, the Asia-Pacific region has experienced a series of large epidemics of HFMD accompanied by abnormal high rates of severe and fatal cases [14–20]. Since then HFMD has started to prompt huge public health concerns in the Asia-Pacific region given its threat to young lives and the potential for its emergence as a leading cause of enterovirus-related CNS disease after poliomyelitis [21].

Given the high prevalence and benign outcome of this common childhood illness, a crucial problem in HFMD prevention and control is how to characterize the differences between mild and severe HFMD cases, so that we can optimize the associated clinical management and public health response. Previous studies have focused principally on their differences in virology, demography, and clinical manifestations [22]. Most of those studies attempt to distinguish severe from mild HFMD cases in early stage which can provide implications for the related clinical management. To the best of our knowledge, few studies have focused on their differences in temporal trends. The temporal trends have played a crucial role in the scientific research of infectious disease, which can be used to inform the mechanism of disease transmission, identify time-varying risk factors, predict disease outbreak, etc. Therefore, obtaining an understanding of the differences and similarities between mild and severe HFMD cases in temporal trends may provide some implications for the HFMD-related public health response. In this study, we conducted a comparative time series analysis based on the surveillance data in mainland China from 2009 to 2014 aiming to compare mild and severe HFMD cases in their temporal trends.

## Methods

### Data source and case definitions

We retrieved the case surveillance data of HFMD from the Chinese Centre for Disease Control and Prevention (China CDC). HFMD was made statutorily notifiable as a Class C infectious disease on 2 May 2008 in mainland China. All clinical cases of HFMD are reported to China CDC via the online reporting system within 24 h of diagnosis by a standardized form.

A clinical case of HFMD is defined as a patient with papular or vesicular rash on hands, feet, mouth or buttocks, with or without fever. A severe case is defined as a clinical case with any CNS complications (including aseptic meningitis, encephalitis, encephalomyelitis, acute flaccid paralysis, or autonomic nervous system dysregulation), or cardiopulmonary dysfunctions (pulmonary edema, pulmonary hemorrhage, or cardiorespiratory failure), or both. Otherwise, the patient is classified as a mild case [23]. More details about case definitions can be found in the Chinese Guidelines for HFMD public health response [24].

We aggregated the daily counts of mild and severe HFMD cases reported between 1 January 2009 and 31 December 2014 based on the date of onset of symptom, respectively. Previous analysis has shown that data collected during the first year are less reliable than those from more recent years, mainly because of improvements in reporting and surveillance [23]. Therefore, we excluded the data collected in 2008 and started our study period from 1 January 2009. Given that the proportion of severe cases is much less than that of mild cases, the huge difference in magnitude (or amplitude) will inevitably tend to mask their relationships in temporal trends. To address above issue, we rescaled both two series by scaling by the arithmetic average for each series separately. The rescaling makes the range of the two series comparable but preserve the temporal trends. We can interpret the values of rescaled time series as the relative daily increase or decrease to the overall mean.

### Statistical analysis

We made the comparisons between the daily series of mild and severe HFMD cases based on the idea of time series decompositions [25]. We assumed each series can be represented as a combination of three components, including the long-term linear trend, periodic variations, and short-term fluctuations. We decomposed each series separately by the application of a quasi-Poisson time series regression model allowing for overdispersion as follows. And then, we compared each of those three components between two series.

$$ Log\left({O}_{it}\right) = {\beta}_{i0}+{\beta}_{il}\times t+{\displaystyle \sum_{j=1}^4}\left({\beta}_{ijs} \sin \left(\frac{2\pi t}{perio{d}_j}\right)+{\beta}_{ijc} \cos \left(\frac{2\pi t}{perio{d}_j}\right)\right)+{\varepsilon}_{it} $$

**Fig. 1:**
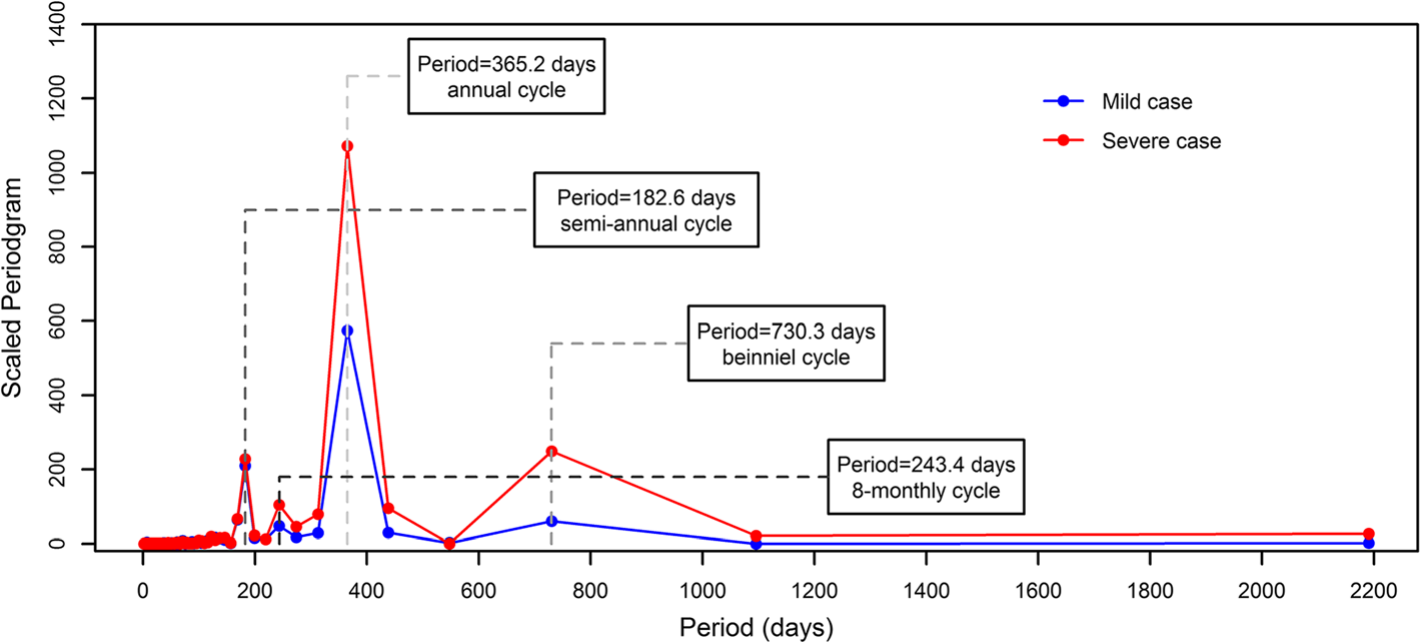
The periodogram of the rescaled daily series of mild and severe HFMD cases. A relatively large value of scaled periodogram indicates relatively more importance for the related cycle in explaining the oscillation in the observed series

*O*_*it*_ is the observed daily series of rescaled mild and severe HFMD cases (denote as *i*, respectively). *β*_*i0*_ is the intercept for series *i. β*_*il*_ is the estimated coefficient of calendar time *t* and the product of them represents the long-term linear trend. The combination of a series of Fourier terms (i.e. sine and cosine functions) with different cycle period *j* represents the periodic variations. The choices of Fourier terms were determined by a preliminary Fourier analysis via plotting the periodogram [26]. We found both series cycled every 2 years and could be characterized by the same four principal cycles, which consist of a semi-annual cycle (period = 182.6 days), an eight-monthly cycle (period = 243.4 days), an annual cycle (period = 365.2 days), and a biennial cycle (period = 730.3 days) (see Fig 1). *ε*_*it*_ represents the short-term fluctuations which are the residuals of the above time series model.

### Comparing the long-term linear trend

As we mentioned earlier, both two series cycled every 2 years. Therefore, the interpretation of the linear predictor of calendar time *t* (i.e. *β*_*il*_, the increase per day) could be misleading. Instead, we calculated the biennial increase of each series based on the estimation of *β*_*il*_, which was given by

$$ BiennialIncreas{e}_i= \exp \left({\beta}_{il}\times 730.3\right)\times 100\%. $$

This figure can be interpreted as the accumulated relative increase in the baseline of the disease incidence over a biennial cycle after adjusting for the periodic variations. Then, we compared the biennial increase between two series and tested their equality (i.e. whether or not the difference between *β*_*il*_ equals zero) by a *Z* test [27] suggested for comparing the slopes between two regressions.

### Comparing the periodic variations

Because the periodic variations consist of four types of cycles, we compared some characteristics of each type of cycles as well as the overall periodic curves between two series. For a particular cycle *j*, we compared its peak value (denote as *P*_*ij*_) and peak timing (denote as *PT*_*ij*_) between two series, which were given by [28]

$$ \begin{array}{l}{P}_{ij}= \exp \left({\upgamma}_{ij}\right)- \exp \left(-{\upgamma}_{ij}\right),\  where\ {\upgamma}_{ij} = \sqrt{{\beta_{ijs}}^2+{\beta_{ijc}}^2}, \\ {}P{T}_{ij}=\frac{1}{2}\times {\mathrm{period}}_j\times \left(1-{\upvarphi}_{ij}/\pi \right),\  where\ {\upvarphi}_{ij} = - \arctan \left({\beta}_{ijs}/{\beta}_{ijc}\right).\end{array} $$

*β*_*ijs*_ and *β*_*ijc*_ are the estimated coefficients of sine and cosine functions for the cycle *j*, *γ*_*ij*_ and *φ*_*ij*_ are the estimated amplitude and phase time for the cycle *j*. For the overall periodic curve, we are unable to derive the parametric forms of its amplitude or phase time because it is a mixture of four types of cycles. Instead, we calculated some of its key characteristics by definitions based on the fitted periodic curve, including the start timing (the temporal position of the minimum value), the peak value (the difference between the peak and minimum value) and the peak timing (the temporal position of the peak value). It could be very cumbersome to derive the parametric distributions of the characteristics that we compared. Therefore, we made the statistical inferences based on the Monte Carol simulations [29]. In simplest terms, we re-generated 1000 replicates of the four types of cycles as well as the overall fitted periodic curves by sampling from the model estimations of the coefficients of Fourier terms (i.e. [*β*_*ijs,*_ *β*_*ijc*_]) assuming a multivariate normal distribution. And then, we obtained the confidence intervals and implemented the statistical tests that we were interested in based on the distributions of the sampled replicates.

### Comparing the short-term fluctuations

Unlike the long-term linear trend and the periodic variations, the short-term fluctuations cannot be expressed as some comparable figures. To compare the short-term fluctuations, we implemented the cross-correlation analysis [30] between two series to see whether they were still temporally related and to explore if there was any potential delay in their temporal trend. Also, we implemented a partial cross-correlation analysis [31] to adjust for the holiday effect (including the summer and winter vacations for school and national public holidays), day of week and the autocorrelations. The selection of the autoregressive terms was informed by the autocorrelation plot for each series.

We did all statistical analysis with R software (version 3.2.3) using the packages *stats* and *TSA*.

## Results

**Fig 2:**
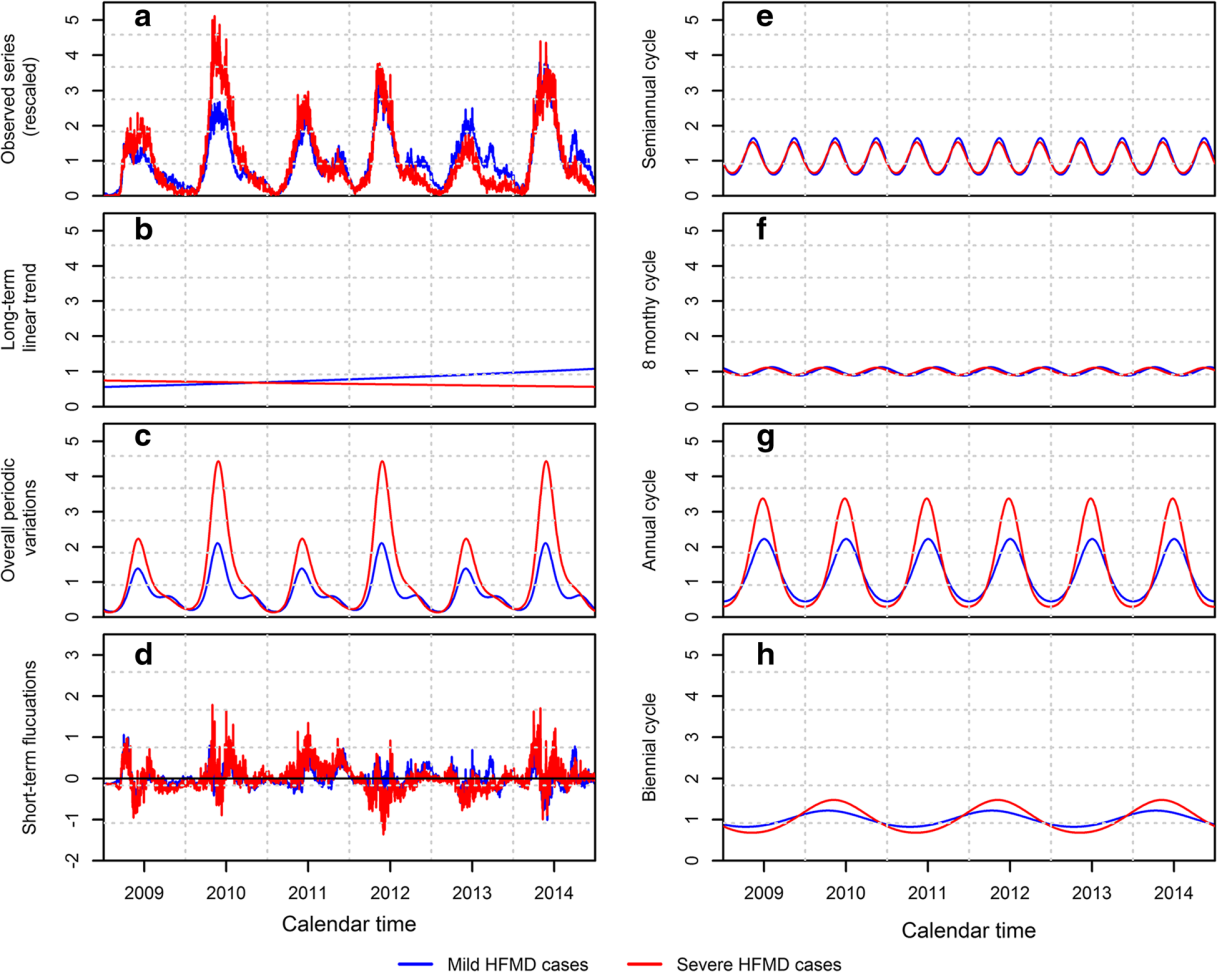
Time series decompositions of the rescaled daily series of mild and severe HFMD cases. **a** the observed series, **b** the estimated long-term linear trend, **c** the estimated overall periodic variations, **d** the short-term fluctuations, **e**–**h** the estimated cyclical components of the overall period variations, including semi-annual cycle, eight-monthly cycle, annual cycle and biennial cycle

In the period of 2009–2014, a total of 11,101,860 clinical HFMD cases together with 115,596 severe cases (accounting for 1.03 % of all clinical cases, while the rest are the mild cases), were reported in mainland China. The daily counts of mild cases ranged from 99 to 19251 with an arithmetic mean of 5067.03 per day. By contrast, the daily counts of severe cases ranged from 0 to 270 with an arithmetic mean of 52.76 per day. After rescaling by the arithmetic average, the daily series of severe cases echoed the patterns seen in the series of mild cases, and both two series showed similar regular variations over time (Fig 2a).

The fitted long-term linear trends of mild and severe cases showed that their baseline of the disease incidence changed in different directions in our study period (Fig 2b). We found a sustained upward trend in the series of mild cases with a biennial increase of 24.46 % (95 % CI: 22.80–26.14 %). Whereas, a downward trend was seen in the series of severe cases with a decrease of 8.80 % (95 % CI: 7.26–10.31 %) every 2 years (Table 1). The difference of the long-term linear trends between two series was statistically significant with a *p*-value less than 0.001.

**Table 1:** The comparisons of time series components between the series of mild and severe HFMD cases

Components	Mild HFMD cases	Severe HFMD cases	ratio/difference^a^	*p*-value*
Long-term linear trend				
Biennial increase (%)	124.46 (122.80, 126.14)	91.20 (89.69, 92.74)	1.36 (1.34,1.39)	<0.001
Semi-annual cycle				
peak value	1.04 (1.00,1.07)	0.87 (0.82,0.93)	1.19 (1.11,1.27)	<0.001
peak time (days)	134.7 (133.77,135.68)	131.48 (129.83,133.13)	3.21 (1.42,5.08)	<0.001
Eight-monthly cycle				
peak value	0.24 (0.20,0.27)	0.21 (0.16,0.26)	1.14 (0.88,1.52)	0.081
peak time (days)	97.47 (91.34,103.51)	74.78 (64.86,83.81)	22.69 (11.03,34.24)	0.001
Annual cycle				
peak value	1.78 (1.73,1.83)	3.08 (2.97,3.19)	0.58 (0.55,0.60)	<0.001
peak time (days)	183.08 (181.79,184,48)	176.52 (175.27,177.77)	6.56 (4.89,8.52)	<0.001
Biennial cycle				
peak value	0.40 (0.36,0.43)	0.80 (0.75,0.86)	0.49 (0.44,0.56)	<0.001
peak time (days)	466.49 (456.14,477.35)	492.55 (483.99,502.11)	−26.06 (−40.96,−11.92)	<0.001
First year cycle of the overall periodic curve				
Start time (days)	36 (35,38)	27 (25,29)	9 (7,11)	<0.001
Major peak time (days)	155 (153,156)	156 (155,158)	−1 (−3,0)	0.012
Major peak value	1.23 (1.19,1.28)	2.10 (2.00,2.19)	0.59 (0.56,0.62)	<0.001
Minor peak time (days)	288 (284,292)	-	-	-
Minor peak value	0.44 (0.42,0.47)	-	-	-
Second year cycle of the overall periodic curve				
Start time (days)	391 (390,392)	377 (375,379)	14 (11,16)	<0.001
Major peak time (days)	510 (509,511)	513 (512,514)	−3 (−4,−1)	<0.001
Major peak value	1.90 (1.85,1.96)	4.20 (4.07,4.34)	0.45 (0.43,0.47)	<0.001
Minor peak time (days)	661 (659,664)	-	-	-
Minor peak value	0.42 (0.36,0.44)	-	-	-

The 95 % confidence interval of model estimates were given in the following bracket

^a^to compare two series, we calculated the relative difference (i.e. the ratio of mild case to severe case) for the biennial increase and the peak value of cycles, whereas the absolute difference (i.e. mild case minus severe cases) was calculated for the start and peak timing. We applied a quasi-Poisson model to estimate the time series components in which a log function is used to link the observed values and linear predictor. Therefore, the ratio of biennial increase and peak value between two series is equivalent to the absolute difference between their related linear predictors. However, the start and peak timing will not be affected by the log link function

*The *p*-value was calculated to test the equality of time series components between two series, with the null hypothesis of no difference (i.e. the ratio equals 1 or difference equals to 0)

As mentioned earlier, the periodic variations of both two series consist of the same four types of cycles (Fig 2e–h). For both two series, the annual cycle made the largest contributions (measured with the size of peak value) to the periodic variations, followed by the semi-annual cycle, biennial cycle and 8-monthy cycle (Table 1). The annual peak value for mild and severe cases were 1.78 (95 % CI: 1.73–1.84) and 3.08 (95 % CI: 2.97–3.19), respectively. We estimated the overall periodic curves by combining the above four types of cycles collectively (Fig 2c). For both two series, the overall periodic curves cycled every 2 years with a high-low pattern (i.e. a relatively low-level epidemic in the first year followed by a high-level epidemic in the next year, denote as first and second year cycle in the following), which was mainly driven by the biennial cycle. Besides, we found two peaks in the annual epidemics of mild cases, including a major peak in early summer and a minor peak in autumn, which were mainly driven by the annual and semi-annual cycle. Whereas, only the major peak was seen in the series of severe cases with a peak value of 2.10 (95 % CI: 2.00–2.19) and 4.20 (95 % CI: 4.07–4.34) for the first and second year cycle respectively, which was nearly twice that of mild cases (Table 1). The discrepancy could be explained by the relatively great peak size of annual and biennial cycle in the series of severe cases, which can conceal the peak of semi-annual cycle. With regards to the peak timing, we found the series of severe cases started the annual epidemic earlier than the series of mild cases with a leading time of 9 (95 % CI: 7–11) and 14 (95 % CI: 11–16) days for the first and second year cycle respectively. However, they reached their major annual peak nearly at the same time (Table 1).

**Fig. 3:**
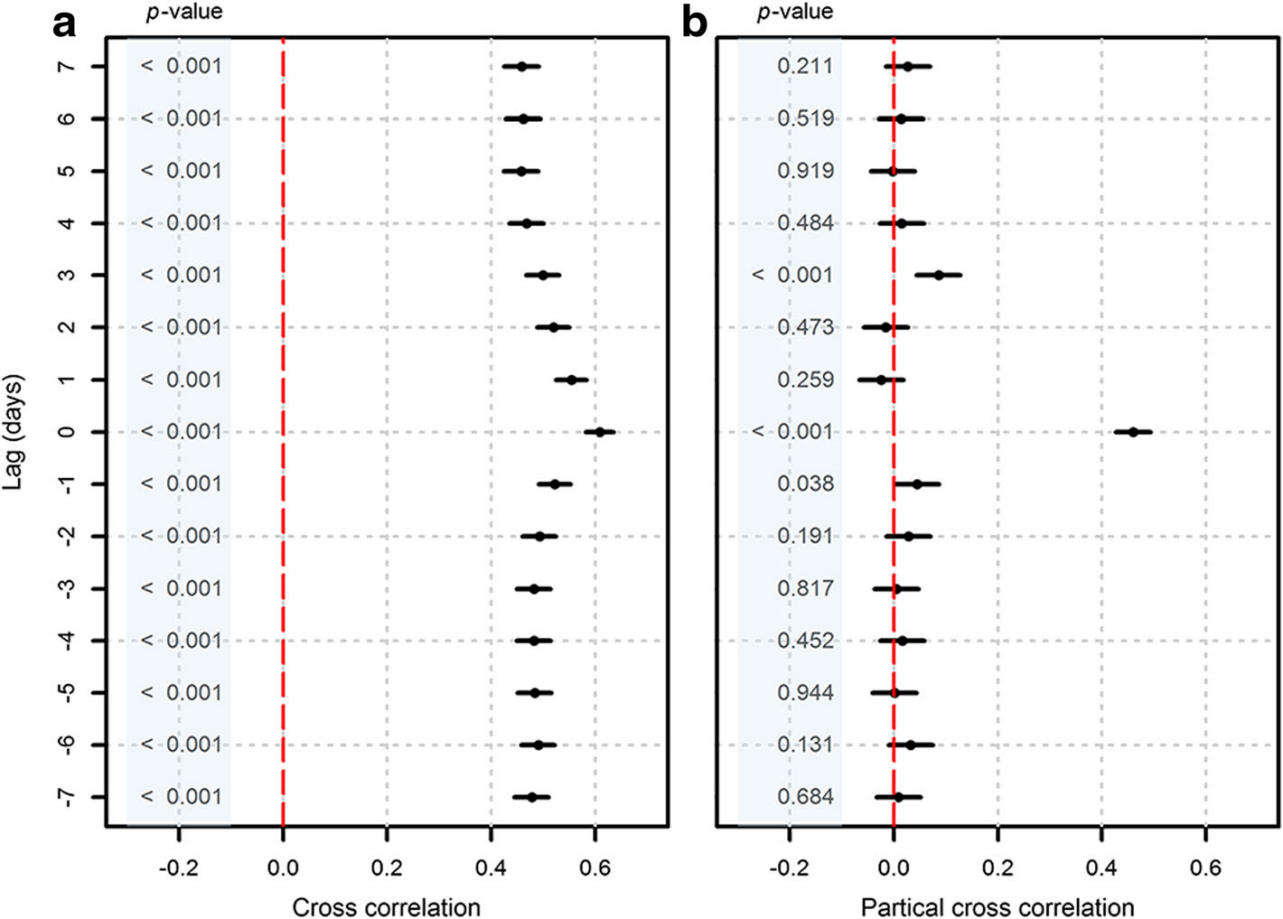
Cross correlations (with 95 % CI) of the short-term fluctuations from lags −7 to +7 days. **a** cross correlation coefficients, **b** partial cross correlation coefficients. The *red dash line* is the reference line of cross correlation coefficient equals to zero. A positive lag means the series of severe cases lag behind the series of mild cases, whereas a negative lag means the series of severe cases lead ahead the series of mild cases

The cross correlation analysis showed that the short-term fluctuations between two series were temporally correlated from lag −7 to +7 days (Fig 3a). We did not detect apparent delayed effects because the highest cross correlation coefficient was obtained at the current day with a value of 0.61 (95 % CI: 0.58–0.63). By further adjusting for the holiday effect, day of week and the autocorrelations, the partial cross correlation coefficient at the current day reduced to 0.46 (95 % CI: 0.43–0.49). Meanwhile, the correlations at other lags became statistically nonsignificant, except a relatively minor effect at the lag of 3 days (Fig 3b).

## Discussions

In this study, we have characterized the temporal trends of HFMD for both the mild and severe HFMD cases. We have found sustained epidemics of HFMD in our study period of 2009–2014. In comparison with reports from other countries or districts in the Asia-Pacific region, the epidemics of HFMD in mainland China exhibited a more regular temporal pattern. In Sarawak (Malaysia) [32] and Japan [33], epidemics of HFMD occurred every 3 to 4 years, while in Taiwan [34], Hong Kong [18] and Singapore [35] the epidemic pattern was more irregular with the inter-epidemic interval varying from 0 to 3 years. The relatively constant growth of population in China [36] could partly explain the regular behaviour of the epidemics in mainland China. Besides, the related vaccines were available until recently after two inactivated monovalent EV-A71 vaccines had been licensed in China in November 2015 [37]. The lack of specific treatments or vaccines could be another reason for the sustained epidemics of HFMD.

Normally, we would expect the baseline of disease incidence (i.e. long-term trend) for a specific infection decreased over time because of the accumulation of immune individuals, just as we saw in the series of severe cases. However, an upward trend was observed in the series of mild cases. Unlike the severe cases which are overwhelmingly caused by EV-A71 [23], the mild symptoms of HFMD can be caused by a wide range of enterovirus. Even though there is evidence of cross-protection between different serotypes of enterovirus, the cross-protection is believed to be temporal [38]. Therefore, the replacement of circulating serotypes of Enterovirus in different years might be one reason for the continued growth of mild cases. Besides, it is worth noting that the number of mild cases is more likely biased by the under reporting compared to that of severe cases because its symptom is benign. According to a retrospective study in Jiangsu province (China) in 2009, only 11.2 % of HFMD cases were reported to the National Infectious Disease Information Management System [39]. Therefore, we cannot rule out the possibility that the upward trend in the series of mild cases may be due just to an improvement in reporting.

By comparing the periodic variations between two series, most of the severe cases were reported around early summer with only one dominant peak every year, whereas the mild cases were more evenly distributed all year around with two annual peaks. Our findings imply that the specific condition in early summer may be more favourable to the transmission of severe cases than any other time of year. The more dominant major peak of severe cases in early summer can lead to a stronger depletion of the pool of susceptible individuals than that of mild cases, which can then partly explain the absence of the minor peak in the following autumn. Consequently, because there is no minor peak in the autumn, we expect a more sufficient replenishment of susceptible individuals for the severe cases in the second half of the year. This is probably why we see an even stronger and earlier major peak in the next year in the series of severe cases. So far, the exact causes of HFMD related severe complications are still unclear [2]. Apart from the infection of EV-A71, previous epidemiological studies suggest that some host factors may also increase the risk of severe complication, such as male gender, young age, etc. [22]. Therefore, the specific condition in early summer may have some impacts on the virulence of EV-A71 or the immunity of hosts. However, further studies are needed to verify our hypothesis.

Even after removing the long-term linear trend and periodic variations, the short-term fluctuations of mild and severe cases have been found to be correlated with each other, especially at the current day. As mentioned earlier, the long-term linear trend and periodic variation of HFMD are mainly driven by some long-term and stable factors, such as the seasonal change of climate and immunity level, the growth of population, the improvement of reporting, etc. However, for the short-term fluctuations of HFMD incidences, it is more likely they are driven by some transient risk factors, for instance, the daily change of temperature and humidity [40, 41]. Our findings suggest that such kind of transient risk factors may have similar impacts on both the mild and severe HFMD cases.

Few previous studies have compared the differences in temporal trends between mild and severe HFMD cases. That is probably because it is very hard to obtain the temporal trends of severe cases given the relatively rare occurrence of severe complications, especially for a small area. We only found one study in Taiwan [42] which examined the temporal associations between the weekly series of mild and severe HFMD cases. It suggested a significantly correlation (r = 0.553) between two types of cases occurring in the same week. Their results are consistent with our findings in the short-term fluctuations. However, the Taiwan study only applied a simple correlation analysis between two series without distinguishing the short-term and long-term temporal trends. As mentioned earlier, the temporal trends at different time scale can be driven by different factors. Therefore, we believe our study can provide a more explicit understanding of HFMD temporal trends.

In this analysis, we did not implement the conventional methods of time series decompositions which was mainly based on the nonparametric smooth function [25]. Instead, we decomposed the time series by the application of a quasi-Poisson regression model. We did this for two main reasons. First, our study interest is different from the conventional purpose of time series decompositions which aim to prediction. In our study, we are more interested in the comparison of temporal trends between two series. By using the regression model, we can extract the parametric forms of temporal trends which allow us to make statistical inferences between two series. Second, the regression form model is more widely used in the field of epidemiological research. Therefore, the methods we used can make our study to be better understood by the potential readers.

There are several limitations in this study. First, we did not conduct any subgroup analyse either by regions or subgroups. As some literature has shown [23, 43–45], the temporal trends of HFMD can vary in different places and subpopulations. Therefore, our conclusions may be not applicable in some specific settings. However, subgroup analyses do raise issues of data sparsity with such a rare occurrence of severe cases. Second, we are unable to analyse the temporal trends of laboratory data in detail to further help with the interpretation of our findings. The laboratory data of HFMD in China were collected on a monthly basis, and so could not provide information at the short-term time scale. Also, the collection of laboratory specimens was not based on the random sampling, and the proportion of laboratory-confirmed cases was very small, hence severely limiting its values in our study [23]. Third, 6 years is still a relatively short period for the time series analysis. As the accumulation of case surveillance data, a longer series would be likely to provide a better and more robust understanding of the temporal trends of HFMD.

## Conclusions

In conclusion, we found some noticeable differences between the daily series of mild and severe HFMD cases in their long-term linear trend and periodic variations, but we also found the short-term fluctuations of these two series were still significant correlated. The different temporal trends between mild and severe HFMD cases can provide us some insights into their potentially different mechanisms of transmission. Our findings can be used to generate hypotheses for future studies to gain a better understanding of the transmission of HFMD, in particular for different types of clinical cases. Besides, the differences between mild and severe cases in temporal trends should also be considered when planning the disease control strategies to optimize the cost-effective. For instance, the more intense major peak in early summer in the series of severe cases may require additional attention when preparing to response the HFMD epidemic.
